# Clinicopathological correlates of Alzheimer’s disease in a general autopsy series from Brazil

**DOI:** 10.1590/S1980-57642008DN10400005

**Published:** 2007

**Authors:** Lea Tenenholz Grinberg, Renata Eloah de Lucena Ferretti, Renata E.P. Leite, Jose Marcelo Farfel, Silmara P. Pacheco, Ana Teresa Di Lorenzo Alho, Renata P. Grisoli, Heidy T.M. Matos, Eliza G. Moreira, Erika S. Balbino, Katia C. Oliveira, Sergio Rosemberg, Heráclito Barbosa Carvalho, Carlos Augusto G. Pasquallucci, Paulo Hilario N. Saldiva, Wilson Jacob-Filho, Ricardo Nitrini

**Affiliations:** 1 Department of Pathology, University of São Paulo Medical School.; 2 Instituto Israelita de Ensino e Pesquisa Albert Einstein, São Paulo.; 3 Division of Geriatrics, University of São Paulo Medical School.; 4 Department of Preventive Medicine, University of São Paulo Medical School.; 5 Behavioral and Cognitive Neurology Unit, Department of Neurology, and Cognitive Disorders Refeence Center (CEREDIC). Hospital das Clínicas of the University of São Paulo Medical School.

**Keywords:** Alzheimer’s disease, dementia, diagnostic criteria, neuropathological criteria, brain bank, doença de Alzheimer, demência, critérios diagnósticos, critérios neuropatológicos, banco de encéfalos, banco de cérebros

## Abstract

The current neuropathological staging models of Alzheimer’s disease (AD) have been developed within the last 20 years. Nevertheless, they were mostly tested on Caucasians of Northern European ancestry or on Asians.

**Objective:**

To verify which of the accepted neuropathologic criteria best discriminates AD from normal aging in a well characterized Brazilian clinicopathological series.

**Methods:**

A random sample consisting of 89 subjects belonging to the Brazilian Brain Bank of the Aging Brain Study were clinically and neuropathologically fully assessed using immunohistochemistry. Clinical and functional statuses were assessed by interviewing a reliable informant. The Clinical dementia rating scale (CDR) was compared to Braak and Braak stage, the consortium to establish a registry for Alzheimer’s disease (CERAD) score and NIA-Reagan (National Institute of Aging - Reagan Institute) score. Subjects with a neuropathologic diagnosis other then AD were excluded (n=27).

**Results:**

The CDR score distribution for the 62 selected subjects was as follows: CDR0=39, CDR0.5=9, CDR1=14. There were no differences regarding age, gender and education among the groups. CDR score correlated best with the CERAD score (r=0.5303; p<0.001) . Braak and Braak stage was significantly higher in subjects with higher CDR. Correlation of the NIA-Reagan criteria was partially disrupted because a large proportion of subjects did not fit any of its categories.

**Conclusions:**

In this series, CERAD criteria better correlated with the CDR groups. Consistent with earlier studies, some cognitively normal subjects have AD neuropathological diagnosis.

## Abstract (pt)

Os modelos de estadiamento neuropatológico da doença de Alzheimer (DA) têm sido desenvolvidos nos últimos 20 anos. Entretanto, têm sido quase exclusivamente testados em caucasianos de ascendência norte-européia ou em asiáticos.

**Objetivos:**

verificar quais dos critérios neuropatológicos discrimina melhor entre a DA e o envelhecimento normal em uma casuística clinicopatológica brasileira bem caracterizada.

**Métodos:**

uma amostra aleatória de 89 casos do Banco Brasileiro de Encéfalos do Estudo de Envelhecimento Cerebral foi submetida à avaliação neuropatológica completa com imunohistoquímica. As condições clínicas e funcionais foram avaliadas mediante entrevista com informante confiável. Os escores na *Clinical Dementia Rating Scale* (CDR) foram comparados com os escores dos estágios de Braak e Braak, do CERAD (*Consortium to Establish a Registry for Alzheimer’s Disease*) e do consórcio NIA-Reagan (National Institute of Aging-Reagan Institute). Casos com diagnósticos neuropatológicos diferentes de DA foram excluídos (n=27).

**Resultados:**

Os 62 casos foram classificados em: CDR0=39, CDR0,5=9, CDR1=14. Não havia diferenças quanto a idade, gênero e escolaridade entre os grupos. Os escores no CERAD correlacionaram-se melhor com os do CDR (*r*=0,5303; *p*<0,001). Os escores nos estágios de Braak e Braak foram significativamente mais elevados nos casos com CDR mais altos. A correlação do CDR com os escores dos critérios NIA-Reagan foi parcialmente rompida porque grande proporção de casos não se enquadrava em nenhuma das categorias diagnósticas destes critérios.

**Conclusões:**

Nesta casuística, os critérios do CERAD correlacionam-se melhor com os do CDR. Como observado por outros estudos, alguns casos de indivíduos cognitivamente normais, preencheram critérios neuropatológicos para o diagnóstico de DA.

Dementia prevalence in Brazil is expected to increase by around 390% between 2001 and 2040.^1^ A definitive diagnosis of Alzheimer’s disease (AD) requires both a clinical history of dementia and neuropathologic confirmation at autopsy. Given that age-related changes overlap with early changes in AD, it is important to adopt neuropathologic criteria able to reliably distinguish between these two conditions. The currently used neuropathologic staging models of AD have been developed in the last 20 years. While the relationship of the maximum frequency of neuritic plaques (NP) with the age of the subject associated to the history of dementia are the pivotal constituents of the Consortium to Establish a Registry for Alzheimer’s Disease criteria (CERAD),^2^ the distribution and burden of neurofibrillary tangles (NFT) determine each of the seven Braak and Braak stages.^3^ Finally, the National Institute on Aging and the Ronald and Nancy Reagan Research Institute of the Alzheimer’s Association^4^ proposed criteria for AD based on both NFT and NP (hereafter called NIA-Reagan criteria). The NIA-Reagan criteria stratifies cases by the likelihood that clinical dementia has been caused by AD lesions in the brain. Table 1 gives a summary of these criteria.

**Table 1:** Summary of the neuropathologic criteria of Alzheimer's disease.

Score name	Stage	Characteristics
Braak and Braak stage (BB)	0 I-II III-IV V-VI	Brain devoid of NFT^†^ NFT^†^ in transentorhinal and entorhinal regions NFT^†^ in the limbic allocortex and adjoining neocortex NFT^†^ in the neocortex, including the secondary and primary fields
CERAD*	Normal	No history of dementia and no NP^‡^
	Possible Probable Definitive	History of dementia and sparse number of NP^‡^ (age-related), or No history of dementia and moderate to severe number of NP (age-related) History of dementia and sparse to moderate number of NP^‡^ (age-related) History of dementia and sparse to severe number of NP^‡^ (age-related)
NIA-Reagan	Normal Low likelihood Intermediate likelihood High likelihood	BB=0 and CERAD= normal BB=I/II and CERAD= possible BB=III/IV and CERAD= probable BB=V/VI and CERAD= definitive

* CERAD: the consortium to establish a registry for Alzheimer's disease;

† NFT: neurofibrillary tangles;

‡ NP: neuritic plaques.

Although more than 10 years have passed since the last criteria were published, which of them best correlates with clinical symptoms remains a matter of debate. In addition, these criteria were largely tested in Caucasians of Northern European ancestry or in Asians^5-7^ and there may be biological differences in burden and distribution of AD changes among different ethnic groups.^8^ The Brazilian population is composed mainly of a mixture of Caucasians of Southern European ancestry, Africans and Indians. The purpose of this study was to examine which of the accepted neuropathologic criteria best discriminate AD from clinical normal aging in a clinicopathological series of 89 subjects belonging to the Brazilian Brain Bank of the Aging Brain Study Group (BBBABSG).

## Methods

A random sample of 89 retrospective cases belonging to the BBBABSG^9^ was studied. Brains were obtained from subjects aged 50 years or older sourced from the Sao Paulo Autopsy Service. Protocols were approved by the local ethics committee and written informed consent forms obtained.

The subjects’ clinical and functional statuses were assessed through a reliable informant. The protocol included a series of semi-structured scales and questionnaires that covered major functional abilities and had been validated for assessment with an informant elsewhere.^9^ Clinical diagnosis of AD and vascular dementia were based on DSMIV-R and NINCDS-ADRDA,^10^ respectively as recommended by the Brazilian Academy of Neurology^11^. The usual criteria were used for other dementias.^12,13^ Neuropathological examinations were carried out based on accepted criteria,^9^ using immunohistochemistry for β-amyloid (4G8), phospho-tau (PHF-1, gift from Peter Davies), α-synuclein (EQV-1, gift of Kenji Ueda) and where required, ubiquitin-1,

Out of the 89 cases, those having a neurodegenerative disease other than AD, or having dementia related to vascular changes were excluded.

The 62 remaining cases were divided into three groups according to the clinical dementia rating (CDR) score:^14^ CDR0=no cognitive decline; CDR0.5=questionable dementia and CDR≥1=dementia. The CDR groups were compared to the Braak and Braak stage, CERAD score and NIA-Reagan score.

Correlations were analyzed using the Spearman’s rank correlation coefficient. The closer *r* is to one, the stronger the correlation. The *r* value was considered statistically significant when p<0.05. Statistical analysis was performed using the software SPSS v. 13 (SPSS, Chicago, Illinois, USA).

## Results

The CDR distribution and demographic data of the 62 selected subjects are described in Table 2. There were no differences regarding age, gender and education among those groups.

**Table 2:** Demographic data of the 62 subjects included in this study.

Cognitive status	Number of subjects	Mean age (in years)	Male:female (%)
CDR 0	39	69.7±12.1	61.5:38.5
CDR 0.5	9	71.6±12.3	66.7:33.3
CDR ≥1	14	82.9±4,9	50:50
Total	62	72.9±12.1	59.7:40.3

Table 3 depicts the distribution of the cases under each CDR group by the CERAD score, Braak and Braak stage and NIA-Reagan score. In 9 subjects, classification according to NIA-Reagan score was not possible due to discrepancies between CERAD score and Braak and Braak stage.

**Table 3:** Distribution of the cases in each CDR group by the CERAD score, Braak and Braak stage and NIA-Reagan score.

		CDR 0	CDR 0.5	CDR ≥1	TOTAL
CERAD score	Normal	29	7	6	42
	Possible	5	0	2	7
	Probable	2	1	2	5
	Definitive	3	1	4	8
Braak and Braak stage	0	18	2	1	21
	I/II	15	4	7	26
	III/IV	5	3	2	10
	V/VI	1	0	4	5
NIA-Reagan score	Normal	29	6	6	41
	Low likelihood	0	1	1	2
	Intermediate likelihood	4	0	2	6
	High likelihood	1	0	3	4
	Criteria not met	5	2	2	9

* CDR: clinical dementia rating.

Table 4 shows the correlation among CDR score and neuropathologic criteria. CERAD score correlated best to the CDR scale (Table 4).

**Table 4:** Clinicopathological correlation by score. The r and p values are depicted on first and second lines, respectively.

Score name	CDR*	CERAD†	Braak and Braak	NIA-Reagan
CDR	1.0000			
CERAD	0.5303 0.0000	1.0000		
Braak and Braak	0.5294 0.0000	0.7563 0.0000	1.0000	
NIA-Reagan	0.5076 0.0002	0.9189 0.0000	0.9102 0.0000	1.0000

* CDR: clinical dementia rating;

† CERAD: the consortium to establish a registry for Alzheimer's disease.

## Discussion

In this series, the CERAD criteria correlated to the CDR score better than Braak and Braak score and NIA-Reagan criteria, although this moderate correlation was only slightly better than for the Braak and Braak score. Results diverge in the literature, with McKeel et al. in 2004^15^ finding NPs to be the best marker to differentiate normal controls from questionable dementia subjects, corroborating previous results,^6,16^ whereas some studies favor the Braak stage for best correlation to dementia severity.^17^

Despite the criteria, majority of studies comparing AD neuropathologic criteria have considered neurofibrillary tangle density and distribution to be directly proportional to severity of AD.^18-20^ The present study corroborates these findings. In our series, 7.7% of the CDR0 subjects met Braak stages ≥ IV, while 43% of the CDR≥ 1 subjects met the same stages.

The casuistic used to correlate NIA-Reagan criteria and CDR was reduced because nine subjects did not meet the criteria in order to be scored. NIA-Reagan criteria demand a perfect match between CERAD score and Braak stage. We are not the first to report problems in using the NIA-Reagan criteria. In 1997, Geddes et al. were only able to classify 22 out of their 47 cases using the NIA-Reagan criteria.^21^ They suggested accommodating discrepant cases having either a probable CERAD score or a limbic Braak and Braak stage, into the intermediate likelihood group of NIA-Reagan criteria. Also, Braak et al. in 1989 published results showing a mismatch between plaques and tangles in staging Alzheimer’s pathology.^22^ Indeed, the r value of CEARD score and Braak and Braak stage correlation was 0.75. Therefore, the highly specific but low sensitivity NIA-Reagan criteria are most used for selecting well defined groups of cases for further analyses. If the prevalence of dementia of the casuistic is low or if the study focuses mainly on control subjects, specificity is usually the most important point and NIA-Reagan criteria are suggested for subject selection.

Perhaps, the current clinical criteria for AD might not be able to detect early changes. Corroborating this hypothesis, neuropathological diagnosis of AD was assigned to control clinical cases in several studies including the present.^23,24^ On the other hand, NP and NFT may be merely markers of AD. Giannakopoulus et al. demonstrated, in a large series of pure AD cases, that more than 50% of CDR scale variability was not explained by NFT or by amyloid deposits.^7^

The present study also has limitations. Informant-based clinicofunctional data might not be reliable enough for detecting early clinical changes. In order to enhance the sensitivity of our interview to early clinical changes, the Informant Questionnaire on Cognitive Decline in the Elderly (IQCODE)^25^ scale is used together with the CDR score. A study performed in Sao Paulo City verified the IQCODE’s high sensitivity and specificity for the local population.^26^ Furthermore, evidence has shown these scales to be reliable even for subjects having mild cognitive impairment.^27^

In sum, this series involving Brazilian samples derived from a general autopsy service corroborates the results found in other clinicopathological series. These preliminary results show that our functional assessment has a good correlation with the neuropathological findings compared to other series. Nevertheless, the clinicopathological correlation is not yet ideal and further studies may reveal better markers and criteria.

## References

[1] Ferri CP, Prince M, Brayne C (2005). Global prevalence of dementia: a Delphi consensus
study. Lancet.

[2] Mirra SS, Heyman A, McKeel D (1991). The Consortium to Establish a Registry for Alzheimer's Disease
(CERAD). Part II. Standardization of the neuropathologic assessment of
Alzheimer's disease. Neurology.

[3] Braak H, Braak E (1991). Neuropathological stageing of Alzheimer-related
changes. Acta Neuropathol.

[4] The National Institute on Aging and Reagan Institute Working Group
on Diagnostic Criteria for the Neuropathological Assessment of
Alzheimer's Disease (1997). Consensus recommendations for the postmortem diagnosis of
Alzheimer's disease. Neurobiol Aging.

[5] Bancher C, Jellinger K, Lassmann H, Fischer P, Leblhuber F (1996). Correlations between mental state and quantitative neuropathology
in the Vienna longitudinal study on dementia. Eur Arch Psychiat Clin Neurosci.

[6] Berg L, McKeel DW, Miller JP (1998). Clinicopathologic studies in cognitively healthy aging and
Alzheimer disease: Relation of histologic markers to dementia severity, age,
sex, and apolipoprotein E genotype. Arch Neurol.

[7] Giannakopoulos P, Gold G, Kovari E (2007). Assessing the cognitive impact of Alzheimer disease pathology and
vascular burden in the aging brain: the Geneva experience. Acta Neuropathol.

[8] Froehlich TE, Bogardus ST Jr., Inouye SK (2001). Dementia and race: are there differences between African
Americans and Caucasians?. J Am Geriatr Soc.

[9] Grinberg LT, Ferretti RE, Farfel JM (2007). Brain bank of the Brazilian aging brain study group - a milestone
reached and more than 1,600 collected brains. Cell Tissue Bank.

[10] McKhann G, Drachman D, Folstein M, Katzman R, Price D, Stadlan EM (1984). Clinical diagnosis of Alzheimer's disease: report of the NINCDS-
ADRDA Work Group under the auspices of Department of Health and Human
Services Task Force on Alzheimer's Disease. Neurology.

[11] Nitrini R, Caramelli P, Bottino CM, Damasceno BP, Brucki SM, Anghinah R (2005). Diagnosis of Alzheimer's disease in Brazil: diagnostic criteria
and auxiliary tests. Recommendations of the Scientific Department of
Cognitive Neurology and Aging of the Brazilian Academy of
Neurology. Arq Neuropsiquiatr.

[12] Neary D, Snowden JS, Gustafson L (1998). Frontotemporal lobar degeneration: a consensus on clinical
diagnostic criteria. Neurology.

[13] McKeith IG, Dickson DW, Lowe J (2005). Diagnosis and management of dementia with Lewy bodies - Third
report of the DLB consortium. Neurology.

[14] Morris JC (1993). The clinical dementia rating (CDR): current version and scoring
rules. Neurology.

[15] McKeel DW, Price JL, Miller JP (2004). Neuropathologic criteria for diagnosing Alzheimer disease in
persons with pure dementia of Alzheimer type. J Neuropathol Exp Neurol.

[16] McKeel DW, Ball MJ, Price JL (1993). Interlaboratory histopathologic assessment of Alzheimer
neuropathology: different methodologies yield comparable diagnostic
results. Alz Dis Assoc Disorder.

[17] Grober E, Dickson D, Sliwinski MJ (1999). Memory and mental status correlates of modified Braak
staging. Neurobiol Aging.

[18] Arriagada PV, Growdon JH, Hedley-Whyte ET, Hyman BT (1992). Neurofibrillary tangles but not senile plaques parallel duration
and severity of Alzheimer's disease. Neurology.

[19] Bierer LM, Hof PR, Purohit DP (1995). Neocortical neurofibrillary tangles correlate with dementia
severity in Alzheimer's disease. Arch Neurol.

[20] Nagy Z, Esiri MM, Jobst KA (1995). Relative roles of plaques and tangles in the dementia of
Alzheimer's disease: correlations using three sets of neuropathological
criteria. Dementia.

[21] Geddes JW, Tekirian TL, Soultanian NS, Ashford JW, Davis DG, Markesbery WR (1997). Comparison of neuropathologic criteria for the diagnosis of
Alzheimer's disease. Neurobiol Aging.

[22] Braak H, Braak E, Ohm T, Bohl J (1989). Alzheimer's disease: mismatch between amyloid plaques and
neuritic plaques. Neurosci Lett.

[23] Morris JC, Storandt M, McKeel DW (1996). Cerebral amyloid deposition and diffuse plaques in ''normal''
aging: evidence for presymptomatic and very mild Alzheimer's
disease. Neurology.

[24] Crystal H, Dickson D, Fuld P (1988). Clinico-pathologic studies in dementia nondemented subjects with
pathologically confirmed Alzheimer's disease. Neurology.

[25] Jorm AF, Jacomb PA (1989). The informant questionnaire on cognitive decline in the elderly
(IQCODE): Socio-demographic correlates, reliability, validity and some
norms. Psychol Med.

[26] Bustamante SE, Bottino CM, Lopes MA (2003). Combined instruments on the evaluation of dementia in the
elderly: preliminary results. Arq Neuropsiquiatr.

[27] Isella V, Villa L, Russo A (2006). Discriminative and predictive power of an informant report in
mild cognitive impairment. J Neurol Neurosurg Psychiatry.

